# Lung cancer diagnosed following emergency admission: a mixed methods study protocol to improve understanding of patients’ characteristics, needs, experiences and outcomes

**DOI:** 10.1186/1472-684X-12-24

**Published:** 2013-05-28

**Authors:** Andrew Wilcock, Vincent Crosby, Sarah Freer, Alison Freemantle, Glenys Caswell, Jane Seymour

**Affiliations:** 1 School of Molecular Medical Sciences, University of Nottingham, Hucknall Road, Nottingham NG5 1PB, UK; 2 Nottingham University Teaching Hospitals NHS Trust, Hucknall Road, Nottingham NG5 1PB, UK; 3 School of Nursing, Midwifery and Physiotherapy, University of Nottingham, Queen’s Medical Centre, Derby Road, Nottingham NG7 2UH, UK

**Keywords:** Carers, Diagnosis following emergency admission, Improving patient experience, Lung cancer

## Abstract

**Background:**

Lung cancer is the leading cause of death from cancer in England. About 40% of patients with lung cancer are diagnosed following an emergency admission (DFEA) to hospital. DFEA is more common in women, and more likely with increasing age and deprivation. Most have advanced disease and survival is poor, but little else is known about this group. The aim of this study is to obtain a detailed understanding of the characteristics, needs, experiences and outcomes of this group.

**Methods/Design:**

This is a single centre study with quantitative and qualitative work packages (WP). WP1 gathers basic details about all patients diagnosed with lung cancer during a 12 month period, focusing on demographics, diagnostic and treatment pathways and selected outcomes. WP2 obtains information from those patients DFEA or, when unable, their carers, about their holistic needs and experiences, using the Sheffield Profile for Assessment and Referral to Care questionnaire and selected questions from the National Cancer Patient Experience Survey. WP3 uses in-depth qualitative interviews with patients and carers to obtain detailed accounts of their symptoms, help-seeking behaviours prior to admission and subsequent experiences of care.

**Discussion:**

Relatively little is known about the experiences of lung cancer patients DFEA and this study will provide detailed information about their needs, characteristics, experiences and outcomes. It should identify areas in the diagnostic and treatment pathway where there is scope to improve the care provided to this group of patients and their carers. The findings will also inform the need for further focused research.

## Background

Lung cancer is the leading cause of death from cancer in England. Prognosis is poor, with one and five year survival rates lower than found in some other countries, in part because of the high proportion of patients presenting with advanced, incurable disease [1,2]. Indeed, about 40% of patients with lung cancer are diagnosed following an emergency admission (DFEA) to hospital, the highest of any cancer. Survival is particularly poor for this group (9% vs. 26% overall at one year) with most having advanced disease. DFEA is more common in women, and more likely with increasing age and deprivation [3,4]. However, relatively little else is known about this group.

This paper presents a study protocol of a research project seeking to identify the characteristics, needs, experiences and outcomes of patients with lung cancer DFEA. In-depth interviews with patients and their informal carers will also explore beliefs, expectations and behaviours prior to diagnosis and what encounters they report with health services prior to the admission.

We anticipate that the study findings will help to identify areas of practice where improvements can be made to improve the outcomes and enhance the experience of patients and their carers.

## Methods/Design

### Study aim and objectives

#### Aim

1. To obtain a detailed understanding of the characteristics, needs, experiences and outcomes of patients with lung cancer who are DFEA to identify areas in the diagnostic and treatment pathway where there is scope to improve the care provided to this group of patients and their carers.

#### Objectives

1. To compare basic demographic details, the steps and time taken through the diagnostic and treatment pathway, and selected outcomes of patients DFEA and those not-DFEA.

2. To obtain from patients DFEA and/or their informal carers, information about their holistic needs and experiences using the Sheffield Profile for Assessment and Referral to Care (SPARC^©^) questionnaire and questions from the National Cancer Patient Experience Survey.

3. To obtain a detailed account of the experience of symptoms and help-seeking behaviours prior to admission and overall care provided by interviewing in depth up to 30 patients DFEA and their carers.

4. To identify areas in the diagnostic and treatment pathway where there is scope to improve the care provided to patients DFEA and their informal carers.

5. To inform the focus of future research.

This is a single centre, prospective mixed methods study. The research setting is a large University Hospital National Health Service Trust in England and the project comprises of three work packages, two using quantitative methods of data collection and one employing qualitative methods. Mixed methods research is that in which ‘the investigator collects and analyses data, integrates the findings, and draws inferences using both qualitative and quantitative approaches or methods in a single study or a program of inquiry’ [5]. Such research takes a pragmatic philosophical and methodological stance in order to address to real world problems.

#### Work package 1

This involves the identification of all patients with lung cancer through the hospital's lung cancer multidisciplinary team meeting (LC-MDT), and collecting information from their hospital records. Over one year, all patients assigned a histological or clinical diagnosis of lung cancer at the two lung cancer multidisciplinary team (LC-MDT) meetings held weekly at the study centre, will be identified by the research team in conjunction with collaborators and the lung cancer nurse specialists and categorised as DFEA or not-DFEA. As quasi-anonymised data, similar to that collected as part of the National Lung Cancer Audit, there is no need to obtain informed consent for this work package [6].

The following data will be collected for all patients: age, gender, ethnicity, performance status (Eastern Co-operative Oncology Group Scale) [7], postcode (recorded as a lower super-output area code to produce a deprivation score), whether seen by a lung cancer nurse specialist at diagnosis or not, diagnostic investigations undertaken (type and timing from referral to secondary care), histological type and stage of lung cancer, time from referral to secondary care to discussion at LC-MDT, treatment recommendation of LC-MDT, treatment received and time from LC-MDT, hospital days utilised, discharge destination, outcome (survival or place of death).

This work package will provide information on the demographics, navigation through the diagnostic and treatment pathway, and outcomes for patients DFEA, and permit comparison with patients who are not-DFEA.

#### Inclusion and exclusion criteria for work packages 2 and 3

All adult patients with lung cancer DFEA are potentially eligible to participate in the questionnaires and the in-depth interview. When a patient is unable to participate, e.g. because of being too unwell, an adult carer will be approached and invited instead. Exclusion criteria are patients and carers who are: experiencing severe distress; who lack capacity to consent or awareness of the diagnosis of lung cancer or are who are unable to communicate in English.

#### Work package 2

All adult patients with lung cancer DFEA identified in work package 1 will be potentially eligible. When the patient is unable to take part, e.g. because of being too unwell, their main carer will be approached instead. After providing an information sheet for a minimum of 24 hours, if they agree to take part, written informed consent will be obtained by a member of the research team. The Charlson co-morbidities index is completed and the patient (or carer) will be asked to complete the Sheffield Profile for Assessment and Referral to Care (SPARC^©^) questionnaire [8-10] and selected items from the 2012 National Cancer Patient Experiences Survey (NCPES) [11]. Questions in the NCPES which relate to information already collected or are irrelevant to this group have been removed. Patients or their carers will only need to complete the questionnaires once.

These data will provide detailed information about the performance status, co-morbidity, holistic needs and experiences of patients who are DFEA. The potential interest of the patient or carer in taking part in the in-depth interview several weeks later (work package 3) is gauged and consent obtained to contact them subsequently.

#### Work package 3

Until recruitment is complete, all patients DFEA and carers who take part in work package 2 will be offered the opportunity to take part in an exploratory qualitative interview, and those indicating a willingness to be involved will receive an information sheet. Subsequently they will be contacted by the Research Assistant and if they agree to take part, written informed consent will be obtained. The qualitative interviews are designed to obtain an understanding of their experiences prior to emergency admission, especially with regards to symptoms and problems experienced. The interview also explores their narratives of the overall care experienced since emergency admission. The interviews will take place several weeks after discharge from hospital. Some patients are likely to die in hospital or shortly after discharge; if so, their informal carers will be approached for interviews at least 6 weeks after bereavement.

We will seek to undertake interviews [12] with patients and carers separately, although some may prefer to be interviewed together. Each interview will be person centred, with questioning taking a flexible format guided by an aide memoire [13]. Participants will be encouraged to recount their experiences in their own terms [14]. Arrangements will be made to follow up issues raised in the interviews, such as unrelieved symptoms or problems in bereavement, with participants’ permission and in collaboration with the responsible clinical teams.

### Sample size

The number of patients diagnosed with lung cancer over a 12 month period will be about 400, with about 150 of these DFEA. This permits an estimate of the precision of any values found in work package 1 when applied to a population level. For the sample sizes of 150 and 250, the two-sided 95% confidence intervals for any observed percentage proportion for any question will not exceed 8 or 6% respectively, when a large sample normal approximation is used [15].

To test for differences in proportions between the two groups, using an alpha of 0.05 we will have 90% power to detect differences of, for example, 10% vs. 22%, 20% vs. 35%, and 30% vs. 46%, and 80% power to detect differences of 10% vs. 20%, 20% vs. 33%, and 30% vs. 44% [16].

A one year period of recruitment should also identify a reasonable number of patients DFEA for work package 2, and ensure that sufficient patients and carers are recruited on time to complete work package 3. Typically, no more than 15 subjects are required to reach data saturation, i.e. no new issues are elicited. Thus, up to 15 patients and 15 carers will be recruited to work package 3.

### Participant withdrawal

It is explained to all potential participants (patients and carers) that entry into the trial is entirely voluntary and that their (or their relative’s) treatment and care will not be affected by their decision. It is also explained that they can withdraw at any time. In the event of their withdrawal it is explained that their data collected so far cannot be erased and we will use the data in the final analyses where appropriate.

### Data analysis

#### Work package 1

The research team will analyse these data using parametric and non-parametric descriptive and comparative statistics as appropriate. Kaplan and Meier survival curves will be compared using the log-rank test. All calculations will be performed using Statistical Package for the Social Sciences (version 18) with a P value of <0.05 considered statistically significant.

#### Work package 2

The research team will analyse these data using appropriate descriptive statistics. All calculations will be performed using Statistical Package for the Social Sciences (version 18).

#### Work package 3

Data collected from the interviews will be analysed by a grounded theory perspective [17], so that attention is focused on the systematic comparison between issues in interviews with patients who are likely to be typical of those DFEA and their carers. Interviews will be recorded, transcribed and entered into a qualitative data analysis package. Coding and thematic analysis will be undertaken by two researchers to check validity. Standard procedures for evaluating rigour in qualitative research will be employed [18].

### Ethical aspects

This study will involve patients and their carers at a stressful time and issues may be raised which require sensitive handling. We will address this through the use of experienced research and specialist nursing staff (Work packages 2 and 3). They will be able to deal with any issues appropriately and ensure that the responsible clinical team are made aware of any need for additional support.

Patient and carer participants completing the questionnaires are advised that the questionnaires have been used and found acceptable by people recently diagnosed with cancer. Nonetheless, they could find some of the questions upsetting or difficult to answer and if this were the case they would not have to answer the question(s). If they are left with any concerns as a result of taking part in the study we would, with their permission, inform the clinical team looking after them so that they could provide them with appropriate support.

Similarly, patient and carer participants taking part in the interviews are advised that they could find some of the questions asked during the interview upsetting or difficult to answer. If this were the case they would not have to answer the question(s). If they were left with any concerns as a result of taking part in the study, with their permission, we would inform the clinical team looking after them so that they could provide them with appropriate support. Further, patient and carer participants in work packages 2 and 3 are made aware that they may withdraw from the study at any time.

Full approval has been obtained from Nottingham 1 Research Ethics Committee, along with the necessary research governance approval from the appropriate Trust.

## Discussion

The aim of this study is to obtain a detailed understanding of the characteristics, needs, experiences and outcomes of patients with lung cancer who are DFEA, and to identify areas in the diagnostic and treatment pathway where there is scope to improve the care provided to this group of patients and their carers.

The findings potentially will have wide ranging implications, involving approaches to achieve earlier diagnosis in primary care to ensuring good end of life care to those admitted with life-limiting complications of advanced disease. The study has several strengths and limitations associated with its design but also challenges, such as the potentially sensitive nature of its focus on an illness with such a high mortality rate.

### Strengths

Relatively little is known about the experiences of lung cancer patients DFEA and, by focusing specifically on this group, this study will provide the most detailed information to date about their needs, characteristics, experiences and outcomes. The research team is comprised of specialist palliative care medical professionals, specialist palliative care nurses, experienced research nurses and social scientists. This mix of disciplines and backgrounds brings a range of perspectives to the conduct of the study and the analyses.

### Limitations

All patients with lung cancer DFEA are eligible to participate in work packages 2 and 3. It is likely, however, that some patients will be too ill to participate and may die soon after diagnosis. Although not ideal, we have tried to mitigate this by inviting a patient’s informal carer to act as a proxy in these circumstances.

## Competing interests

The authors declare that they have no competing interests.

## Authors’ contributions

GC wrote the first draft of the paper based on a study protocol designed by AW and all other authors and submitted to the Roy Castle Lung Foundation. JS and AW revised the draft and all other authors agreed its content. All authors read and approved the final manuscript.

## Pre-publication history

The pre-publication history for this paper can be accessed here:

http://www.biomedcentral.com/1472-684X/12/24/prepub
